# Multiplexed Massively Parallel Sequencing of Plastomes Provides Insights Into the Genetic Diversity, Population Structure, and Phylogeography of Wild and Cultivated *Coptis chinensis*

**DOI:** 10.3389/fpls.2022.923600

**Published:** 2022-07-07

**Authors:** Yiheng Wang, Jiahui Sun, Zhenyu Zhao, Chao Xu, Ping Qiao, Sheng Wang, Mengli Wang, Zegang Xu, Qingjun Yuan, Lanping Guo, Luqi Huang

**Affiliations:** ^1^National Resource Center for Chinese Materia Medica, China Academy of Chinese Medical Sciences, Beijing, China; ^2^State Key Laboratory of Systematic and Evolutionary Botany, Institute of Botany, Chinese Academy of Sciences, Beijing, China; ^3^Academician Workstation, Jiangxi University of Traditional Chinese Medicine, Nanchang, China; ^4^Lichuan Jianzhuxi Huanglian Cooperative, Lichuan, China

**Keywords:** *Coptis chinensis*, wild and cultivated, genetic diversity, phylogeography, population structure, massively parallel sequencing, plastome

## Abstract

Root rot has been a major problem for cultivated populations of *Coptis chinensis* var. *chinensis* in recent years. *C. chinensis* var. *brevisepala*, the closest wild relative of *C. chinensis* var. *chinensis*, has a scattered distribution across southwestern China and is an important wild resource. Genetic diversity is associated with greater evolutionary potential and resilience of species or populations and is important for the breeding and conservation of species. Here, we conducted multiplexed massively parallel sequencing of the plastomes of 227 accessions of wild and cultivated *C. chinensis* using 111 marker pairs to study patterns of genetic diversity, population structure, and phylogeography among wild and cultivated *C. chinensis* populations. Wild and cultivated resources diverged approximately 2.83 Mya. The cultivated resources experienced a severe genetic bottleneck and possess highly mixed germplasm. However, high genetic diversity has been retained in the wild resources, and subpopulations in different locations differed in genotype composition. The significant divergence in the genetic diversity of wild and cultivated resources indicates that they require different conservation strategies. Wild resources require *in situ* conservation strategies aiming to expand population sizes while maintaining levels of genetic diversity; by contrast, germplasm resource nurseries with genotypes of cultivated resources and planned distribution measures are needed for the conservation of cultivated resources to prevent cultivated populations from undergoing severe genetic bottlenecks. The results of this study provide comprehensive insights into the genetic diversity, population structure, and phylogeography of *C. chinensis* and will facilitate future breeding and conservation efforts.

## Introduction

Deterministic or stochastic forces, such as natural selection and genetic drift, increase genetic diversity at several levels of biological organization (individuals, populations, and species) (Sun et al., 2021). Higher genetic diversity generally results in more adapted genotypes and is associated with greater evolutionary potential and resilience of species or populations (Falk and Holsinger, 1991). Low genetic diversity can compromise the resistance of species to abiotic and biotic stress (Varshney et al., 2019). An understanding of genetic diversity is critically important for identifying morphologically indistinguishable species, reconstructing the phylogeographic history of lineages, and managing biodiversity (Nevo, 2001). Phylogeographic studies can provide insight into the demographic, evolutionary history, and structure of populations or young lineages (populations or closely related species) based on genetic and geographic data, and this information has breeding and conservation implications (Avise, 2000; Hickerson et al., 2010).

Plastomes have been extensively used in population diversity analyses and phylogeographic studies because of their small size, low rate of recombination and molecular evolution, and uniparental inheritance pattern (Dong et al., 2021a,b,c; Wang et al., 2021). With the advent of next-generation sequencing technologies, plastids can now be sequenced and assembled at a relatively low cost. However, sequencing plastids efficiently becomes impractical when sample sizes range from hundreds to thousands of individuals. One approach for overcoming this problem is whole plastid sequencing combined with massively parallel sequencing (MPS). Inverted repeat regions (IRa, IRb) of plastids are highly conserved, and most variable sites are located in the small single-copy region (SSC) and large single-copy region (LSC). At the population level, coding regions are more conserved compared with spacer regions, and nucleotide diversity varies among genes. Thus, the many regions with few variable sites in plastids do not provide sufficient data for conducting diversity and phylogeographic analyses at the population level. Comparison of representative plastids, identification of variable regions, and the development of primer markers for MPS is a more efficient approach than direct sequencing of individual plastids.

*Coptis* is a perennial herbaceous genus of the Ranunculaceae family that has been used in traditional Chinese medicine for its antibacterial and anti-inflammatory properties for thousands of years (Liu et al., 2021a). In China, all members of the genus have been designated as national second-class endangered plants. Indigenes in China have utilized various *Coptis* species as herbal medicines, and three of them, *C. chinensis* var. *chinensis* “Weilian,” *C. deltoidea* “Yalian,” and *C. teeta* “Yunlian,” are classified as official Huanglians in the Chinese Pharmacopeia.

The area of *C. deltoidea* and *C. teeta* cultivation is small; however, *C. chinensis* var. *chinensis* is cultivated over a wide area in the provinces Chongqing, Sichuan, Hubei, and Hunan (0.32 million mus), and has been cultivated since the Yuan era (ca. 700 years) (Wang et al., 2020). The historically intensive harvesting of herbs, especially wild resources, which was motivated by the belief that wild resources provided superior benefits compared with cultivated resources, resulted in the extinction of wild *C. chinensis* var. *chinensis* resources in recent decades. Consequently, all *C. chinensis* var. *chinensis* material used in this study was derived from cultivated *C. chinensis* resources.

*Coptis chinensis* var. *brevisepala*, the closest wild relative of *C. chinensis* var. *chinensis*, exhibits a scattered distribution across southwestern China. It has long been cited as an excellent Huanglian [e.g., in Ben-Cao-Tu-Jin (1061 A.D.) and Ben-Cao-Gang-Mu-Shi-Yi (1765 A.D.)] (Peng et al., 2017). The area of farmland in southwestern China has increased due to the rapid growth of the human population in the last century, and this resulted in a reduction in suitable *C. chinensis* var. *brevisepala* habitat. The decline of wild populations and the slow growth of wild plants, coupled with the high yield of cultivated, led to a decline of wild *C. chinensis* var. *brevisepala* resources in the traditional Chinese medicine market. Despite this decline in the last century, its high utility and economic value still make *C. chinensis* var. *brevisepala* a desirable wild *C. chinensis* resource. Conservation measures thus need to be taken to prevent further declines in *C. chinensis* var. *brevisepala* populations.

Root rot has been a major problem for cultivated *C. chinensis* in recent years. Domestication of *C. chinensis*, as in many cultivated plants, may result in genetic bottlenecks and decrease variation in disease resistance within populations (Varshney et al., 2019). Extrinsic factors include large-scale general agricultural practices deleterious to the maintenance of ecological balance, irregular planting practices such as over-fertilization, and the excessive application of pesticides. Wild *C. chinensis* populations that have not been subject to artificial selection and domestication have high levels of genetic diversity by comparison. These wild resources are important reservoirs of genetic diversity that should be conserved, as stress-resistant individuals could be used to improve *C. chinensis* germplasm. Characterizing patterns of genetic diversity in cultivated and wild resources and clarifying phylogeographic relationships among different populations are essential for identifying valuable genotypes in selective breeding programs and developing germplasm conservation strategies (Zhou et al., 2015). However, robust evaluations of the genetic diversity and structure of wild and cultivated resources require large samples sizes and geographically thorough sampling.

Here, we used a representative set of plastids and MPS data of members of the genus Coptis to (1) clarify evolutionary relationships within *Coptis* distributed in China, (2) elucidate the genetic diversity of wild and cultivated *C. chinensis* and their phylogeographic relationships through the rapid and cost-efficient MPS approach, and (3) propose germplasm conservation strategies for wild and cultivated *C. chinensis* resources.

## Materials and Methods

### Plant Material, DNA Extraction and Sequencing

A total of 23 *Coptis* species were collected for plastid sequencing, and the 227 accessions used for MPS were collected across the entire distribution of *Coptis*; four plastids were downloaded from GenBank with accession numbers MT773635–MT773638 (Supplementary Tables 1, 3). Total genomic DNA was extracted from fresh leaves of a single individual using a modified CTAB method and purified using a Wizard DNA clean-up system (Promega, Madison, WI, United States) (Li et al., 2013). All the DNA and molecular material were deposited in the herbarium of the Institute of Chinese Materia Medica (CMMI). PE150 sequencing was conducted to sequence plastids on an Illumina HiSeq XTen platform at Novogene (Tianjin, China). A four-step approach was used to construct the amplicon library for MPS. First, primers were designed with 400-bp targeted regions by Se-al software to cover nearly all variable regions of *C. chinensis* plastids. Second, we amplified all the 227 accessions with 111 primer pairs using the LGC High-throughput workstation system in the Maize Research Center, Beijing Academy of Agriculture and Forestry Sciences. Third, labeled PCR was performed to attach sample-specific oligo-tags to distinguish samples using the products from the second step as the template. A paired-end library with mixed labeled-products was constructed using a NEBNext UltraTM DNA library prep kit and sequenced at Novogene (Tianjin, China) on an Illumina HiSeq2500 platform (PE 250 sequencing). Procedures for MPS library construction followed the protocol of Liu et al. (2021c).

### Plastome Assembly and Annotation

The raw sequencing reads of the PE150 data were qualitatively controlled by Trimmomatic v0.39 for filtering primer/adaptor sequences and low-quality reads to obtain high-quality reads (using settings: ILLUMINACLIP:TruSeq3-PE.fa:2:30:10:1:true LEADING:20 TRAILING:20 SLIDINGWINDOW:4:15) (Bolger et al., 2014). GetOrganelle v1.7.5 was used for the *de novo* assembly of the high-quality reads with following settings: -F embplant_pt, -R 15 and -K 105 (Jin et al., 2020). And Geneious v8.1, a double-check process, was used to map all high-quality reads to the assembled plastome sequence to verify the assembly accuracy (He et al., 2014; Dong et al., 2022). Ambiguous regions and four junctions between IRs and SCs in the plastid were confirmed manually. Gene annotation was performed using the online platform CPGAVAS2 using a build-in database with 2,544 plastids as reference (Shi et al., 2019). If necessary, the positions of the start and stop codons and boundaries between introns and exons were manually corrected in Sequin. The annotation results were further checked using Geneious v8.1. The circle plastid map was drawn using the online program Chloroplot (Zheng et al., 2020).

### Amplicon Data Quality Control and Consensus Generation

Low-quality sequences and sequences shorter than 200 bp were removed from the PE250 dataset using the NGS QC toolkit v2.3.3 with default settings (Patel and Jain, 2012). The clean data were demultiplexed with the FASTX-Toolkit v0.0.13^1^ using sample-specific oligo-tags and primers. Finally, large consensus sequences of different primers and samples were generated using Cotu-Generator.py (https://github.com/YanleiLiu1989/Cotu-master). Procedures of the amplicon data processing followed the protocols of Liu et al. (2021c).

### Phylogeny and Divergence Time Estimation

A total of 27 *Coptis* plastids were aligned using the MAFFT v7 online service with the “auto” strategy and manually adjusted using MEGA X, which produced dataset-I (Kumar et al., 2018; Katoh et al., 2019). The program ModelFinder was used to select the best-fit model (TVM+F+R2) based on the BIC criterion (Kalyaanamoorthy et al., 2017). The maximum likelihood tree was inferred using IQ-TREE with the TVM+F+R2 model and 5,000 ultrafast bootstraps (Zhang et al., 2020); the phylogeny was displayed in FigTree v1.3.1.

Divergence time was estimated using BEAST v2.6.6 with the following parameter settings: Relaxed Log Normal clock model, Yule speciation model, and GTR substitution model (Bouckaert et al., 2019). The split times of *C. quinquesecta* (6.53 Mya) and *C. japonica* (4.85 Mya) estimated by Xiang et al. (2018) were used as secondary calibration points. The Markov chain Monte Carlo chains (MCMC) were run for 500,000,000 generations and sampled every 1,000 generations. The effective sample size (ESS) was checked using Tracer v1.6 to ensure that all parameters exceeded 200. The first 25% of runs were discarded as burn-in. TreeAnnotator v2.6.6 was used to produce a maximum clade credibility (MCC) tree. Divergence time with 95% HPD intervals was displayed using FigTree v1.3.1 and modified in AI CS6.

### Comparative Analysis of Plastomes

Data on genome size, GC content, the sizes of the four regions of the plastid, and the number of genes were summarized in Geneious. The variable sites, haplotypes, haplotype diversity (Hd), indels, and Pi of 10 *C. chinensis* var. *chinensis* accessions and 12 *C. chinensis* var. *brevisepala* accessions were calculated using DnaSP v5.10 (Librado and Rozas, 2009). The indels were counted with the gap option “Multiallelic;” the number of indels was also manually counted in every 500-bp sliding window. Pi of every 500-bp sliding window was also calculated using DnaSP v5.10. Circos analysis was performed on the indel and Pi data using OmicStudio tools at https://www.omicstudio.cn/tool/.

### Genetic Relationships, Differentiation, and Structure of Subpopulations

We composed a supermatrix (dataset-II) using SequenceMatrix v1.7.8 to concatenate 25,197 cpDNA fragments generated by MPS (227 accessions, 111 primer pairs) (Vaidya et al., 2011). For dataset-II, haplotype data were analyzed in DnaSP v5.10, and haplotype frequencies in populations were calculated using Arlequin v3.5.1.3 for subsequent network analysis (Excoffier and Lischer, 2010). A TCS network was built using PopArt v1.7, and four *C. omeiensis* accessions were used as outgroups (Clement et al., 2000; Leigh and Bryant, 2015).

Dataset-III was generated by discarding four outgroups. A submatrix containing 917 SNPs was generated in DnaSP v5.10, and sites with alignment gaps were included if a polymorphism was present. The data were converted into a suitable format using GenAlEx v6.5 and then used for STRUCTURE and PCA. The R package “PCAtools” was used to conduct PCA and make PCA plots. The STRUCTURE workflow was as follows: (1) the optimal number of clusters was determined by running the K-means clustering algorithm from *K* = 2 to *K* = 10 with ten runs for each K-value, (2) the initial burn-in period was set to 10,000 with 10,000 MCMCs, (3) the most suitable clusters were determined using the DeltaK method on Structure Harvester^2^ and aggregated by running the CLUMPP program (Jakobsson and Rosenberg, 2007), and (4) plots were built using the R package “ggplot2,” To quantify the degree of differentiation among populations, AMOVA and pairwise Fst calculations were conducted in Arlequin v3.5 using default settings.

## Results

### Characteristics of the Plastid

**FIGURE 1:**
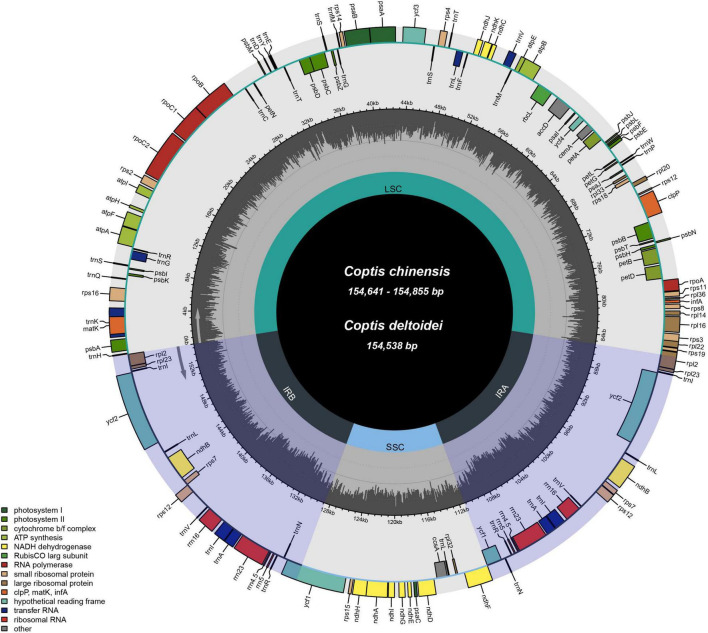
Gene maps of the plastids of *C. chinensis* and *C. deltoidea*. The genes are color-coded based on their functions. The dashed area indicates the GC composition of the plastid.

We obtained 23 complete plastids by *de novo* assembly of 22 *C. chinensis* accessions [10 *C. chinensis* var. *chinensis* (Ccc) accessions and 12 *C. chinensis* var. *brevisepala* (Ccb) accessions] from different provinces in China and one *C. deltoidei*, as well as four other related species from GenBank. All 27 plastids had a typical quadripartite structure comprising two IR regions (26,154–26,225 bp) separated by the LSC (84,240–85,168 bp) and the SSC (17,232–17,550 bp), and the genome size ranged from 154,156 bp (MT773638) to 154,985 bp (MT773635). *Coptis* plastids were highly conserved in GC content and the number of genes. The total GC content ranged from 38.1 to 38.2%. In addition, all the sequences contained 80 protein-coding genes, 29 tRNA genes, and four rRNA genes (Figure 1 and Supplementary Table 1). The 27 plastids yielded dataset-I (aligned size of 156,497 bp) and the nucleotide diversity (Pi) was 0.00282 (Supplementary Table 2).

### Plastid Variation and Development of Marker Pairs

**FIGURE 2:**
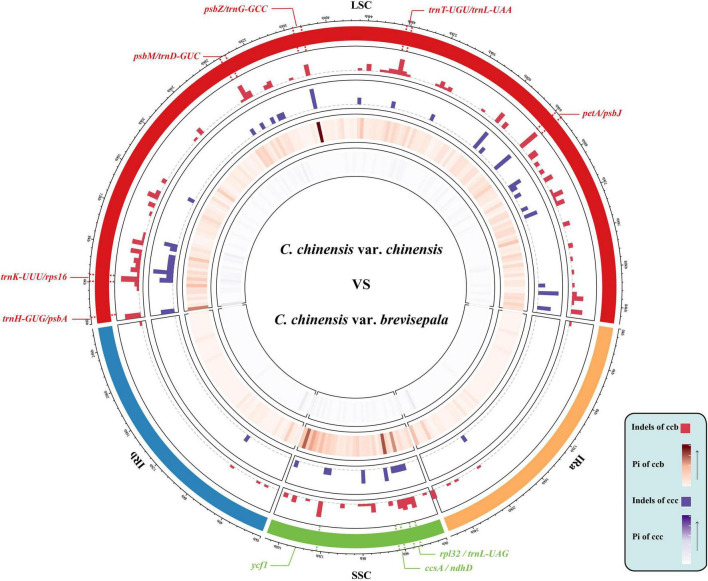
Circos plot showing the indel and nucleotide diversity of wild and cultivated *C. chinensis*. The concentric circles from outer to inner indicate the following: quadripartite structure of the plastid represented by different colors; the indels of Ccb; the indels of Ccc; the nucleotide diversity of Ccb; and the nucleotide diversity of Ccc. All statistics were computed for windows of 500 bp. In the Ccb population, Pi varied from 0 to 0.01442, and the average Pi was 0.00137. In the Ccc population, Pi ranged from 0 to 0.00569, and the average Pi was extremely low (approximately 0.00026).

The 22 aligned sequences were 155,489 bp in length. We recovered 926 variable sites, 12 haplotypes, and 334 indels, and the average Pi was 0.00197. The Ccc population had 107 variable sites, and five haplotypes with a diversity (Hd) of 0.822. The Ccb population had 596 variable sites, seven haplotypes, and 222 indels, and Hd was 0.924 (Table 1). Based on sliding window analysis in DnaSP, Pi and indels were visualized in a Circos map (Figure 2). The regions with higher Pi and high indel counts were mostly located in the LSC and SSC, and only a small portion was distributed in the junction of the IR regions. The variable regions of Pi were consistent with those of indels, and the Pi and indel counts were statistically higher in the Ccb population (Supplementary Figure 1). Nine hot spots were observed in the most outer circle, which were determined based on whether the indel count was greater than seven and the Pi was higher than 0.006 (*rpl32-trnL^UAG^*, *ccsA-ndhD*, *ycf1*, *trnH-psbA*, *trnK^UUU^-rps16*, *psbM-trnD^GUC^*, *psbZ-trnG^GCC^*, *trnT^UGU^-trnL^UAA^*, and *petA-psbJ*). The region of *petA-psbJ* contained the greatest number of indels and the highest value of Pi. Overall, the results in the Circos map indicated that the Ccb population was diverse. The marker pairs covered all the variable sites including SNPs and indels were developed for MPS. After the validation step, 111 marker pairs were obtained and used for population analyses.

**TABLE 1:** Chloroplast genome diversity of *C. chinensis* populations.

Population	Number of accessions	Aligned length	Variable sites	Nucleotide diversity (Pi)	Number of Hap	Hap diversity	Number of indels
Ccc	10	155,034 bp	107	0.00026	5	0.822	58
Ccb	12	155,198 bp	596	0.00137	7	0.924	222
Total	22	155,489 bp	926	0.00197	12	0.952	334

### Phylogenetic Reconstruction and Divergence Time Estimation

**FIGURE 3:**
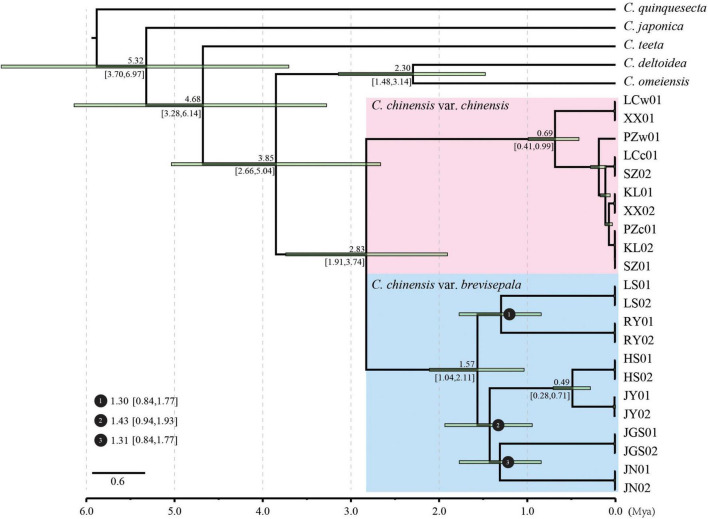
Divergence time estimation based on the plastids of *Coptis*. Numbers above and under the branches indicate the mean divergence times and 95% confidence interval of each node, respectively. Green bars indicate the 95% highest posterior density intervals. Divergence time and the timeline are indicated in million years ago (Mya).

A phylogenetic analysis of dataset-I was conducted using IQ-tree methods. The best-fit model was TVM+F+R2 according to the Bayesian information criterion (BIC) (Supplementary Table 4). Molecular clock analysis was performed to estimate the divergence time of *Coptis* species in China. The relationships among all taxa were well resolved and fully supported, suggesting that the plastids provided increased resolution for phylogenetic reconstruction (Figure 3 and Supplementary Figure 2). Accessions of *C. chinensis* formed a monophyletic group with 100% bootstrap support that was sister to a clade containing *C. deltoidea* and *C. omeiensis*. Two highly supported clades within *C. chinensis* corresponding to Ccc and Ccb were clearly separated. Ten accessions of Ccc generated two major subclades that were not congruent with their geographic distributions. However, 12 accessions of Ccb formed six subclades that were congruent with their geographic distributions. Our divergence time analysis revealed that the divergence of *C. chinensis* from its closest relative occurred 3.85 Mya (95% HPD: 2.66–5.04 Mya). Ccc and Ccb split apart from each other approximately 2.83 Mya (95% HPD: 1.91–3.74 Mya). In addition, diversification of the branches representing different subpopulations within Ccb mostly occurred over a short period ranging from 1.30 to 1.57 Mya; however, the HS and JY subpopulations split recently at approximately 0.49 Mya.

### Genetic Differentiation of Wild and Cultivated Populations

Large consensus sequences of 227 accessions with 111 marker pairs comprised dataset-II, which was 43,354 bp (aligned size) and had a Pi of 0.00305. Dataset-III was a subset of dataset-II without the four outgroups that was 43,283 bp (aligned size) and had a Pi of 0.00293 (Supplementary Table 2). Pi was higher for dataset-II and III, and the sequence sizes of these datasets were shorter (by approximately 75%) than those in dataset-I.

**FIGURE 4:**
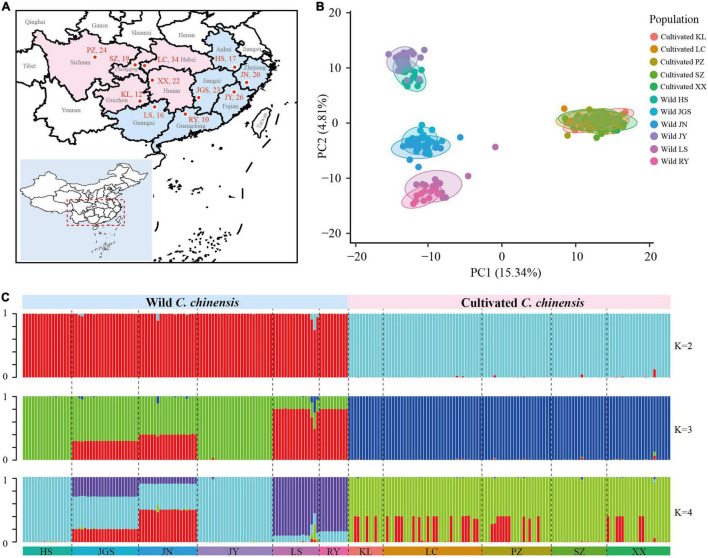
Population structure of *C. chinensis* populations. **(A)** Geographic distribution of the sampling locations. The map plot was generated using an R package (https://github.com/linhesun/bilibiliRlearning/tree/master/2021_r21_china_map). **(B)** PCA of wild and cultivated populations; the proportion of the variance explained was 15.34% for PC1 and 4.81% for PC2. **(C)** STRUCTURE analysis for *K* = 2–4. Colors indicate different clusters. The x-axis shows the subpopulations, and the y-axis indicates the probability of inferred ancestral lineages.

**FIGURE 5:**
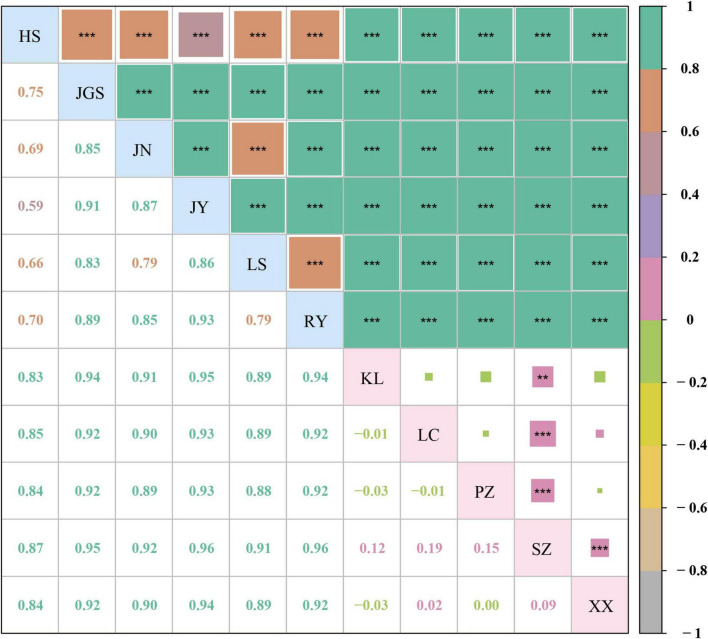
Pairwise Fst values among 11 subpopulations of *C. chinensis*. The letters on the diagonal represent 11 subpopulations of *C. chinensis*. Below the diagonal, the number in the box is the pairwise Fst value between a vertical subpopulation and a horizontal subpopulation. Above the diagonal, pairwise Fst value is reflected by area and color of the square. The higher the Fst value is, the larger the colored area is. The color referring to the Fst value range is indicated in the column chart on the far right. The *P*-value is the probability calculated *via* significance test. ** indicates *P* < 0.01, *** indicates *P* < 0.001.

In dataset-III, 223 accessions were arranged in 11 subpopulations (six Ccb and five of Ccc) that were congruent with their geographic distributions (Figure 4A). The genetic differentiation between wild and cultivated subpopulations was estimated using Fst (Figure 5). The Fst heatmap revealed that wild populations were markedly divergent from cultivated populations (Fst > 0.83). The Fst values ranged from −0.03 to 0.19 among cultivated subpopulations, and the “SZ” subpopulation was the only cultivated subpopulation that was divergent from the other cultivated subpopulations. However, little differentiation was observed among the remaining cultivated subpopulations. Fst values for the wild subpopulations ranged from 0.59 to 0.93, indicating that the wild subpopulations were highly differentiated. Analysis of molecular variance (AMOVA) revealed significant genetic differentiation among Ccb and Ccc populations, indicating that most of the genetic diversity of Ccc existed within populations (95.05%, *P* < 0.01), whereas most of the genetic diversity of Ccb existed among populations (80.87%, *P* < 0.001) (Table 2).

**TABLE 2:** Analyses of molecular variance (AMOVA) of Ccc and Ccb.

Group	Source of variation	Percentage of variation (%)
Ccc	Among populations	4.94*
	Within populations	95.05*
Ccb	Among populations	80.87**
	Within populations	19.13**

*P < 0.01 and **P < 0.001.

### Genetic Structure of Wild and Cultivated Populations

To visualize the genetic structure of wild and cultivated populations, principal component analysis (PCA) was performed using the 917 SNPs from dataset-III. PCA revealed marked separation between the wild and cultivated subpopulations (Figure 4B). Cultivated subpopulations were clustered into a single group, whereas wild subpopulations were clustered into three groups: HS/JY, JGS/JN, and LS/RY. STRUCTURE analysis was conducted to characterize the genetic structure among subpopulations (Figure 4C). The largest delta K value was observed for *K* = 2, followed by *K* = 3 and *K* = 4 (Supplementary Figure 3), which indicated that the division of the 223 accessions into two populations received the strongest support. According to the STRUCTURE results, the distinction between wild and cultivated subpopulations could be observed regardless of whether K was 2, 3, or 4, indicating that little gene flow has occurred between wild and cultivated groups. In the cultivated group, patterns of genetic structure inferred by the STRUCTURE analysis were not congruent with the geographic distribution of subpopulations, indicating germplasm mixture. In the wild group, patterns were congruent with the geographic distribution of subpopulations; although there was some gene flow between subpopulations, germplasm diversity was high among subpopulations but low within subpopulations. Based on the patterns of diversity among subpopulations, HS was more genetically similar to JY and so as JGS to JN, LS to RY.

**FIGURE 6:**
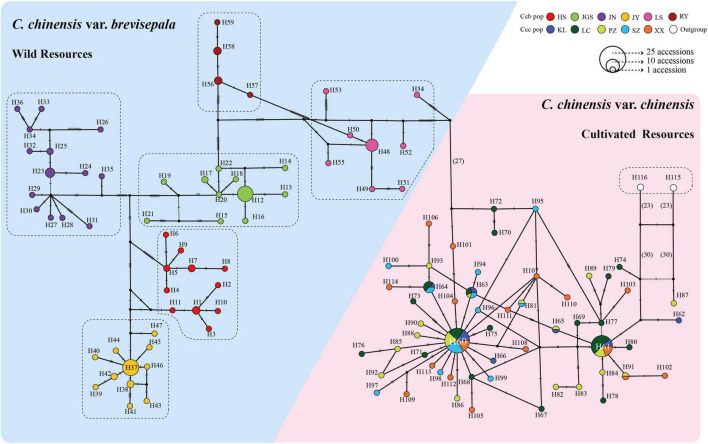
TCS network for all 227 accessions based on 25,197 cpDNA fragments. Circle size is proportional to haplotype frequency. Different colors indicate different subpopulations from different distributions, and missing haplotypes are indicated by black dots. Mutational steps are indicated by hatchures (if the number of steps is less than 20) and number (if the number of steps is greater than 20).

A TCS network based on 116 haplotypes (H115 and H116 of *C. omeiensis* were used as the outgroup) indicated that wild and cultivated populations formed distinct groups; the clustering of wild accessions was congruent with their geographic distributions; and cultivated populations were highly admixed (Figure 6). Overall, the phylogeographic structure of the subpopulations was consistent with the Fst calculations and the AMOVA results.

## Discussion

### Applicability and Economy of the Massively Parallel Sequencing Approach

Sequencing can be cost-prohibitive, and whole-genome sequencing is often not necessary. Samples sizes are typically medium to large in population, phylogenetic, and phylogeographic research, and variable regions are more important than genome structure, gene order, and genetic composition. MPS can be used to acquire information on the variable regions from accessions. Using a specific library preparation, we can sequence multiple regions and hundreds of accessions in a MPS run (Parks et al., 2009). The applications of MPS extend beyond phylogenetic and phylogeographic studies, as MPS has also been used in forensics, genetic medicine, and environmental studies (Tucker et al., 2009; Lopes et al., 2017; Bruijns et al., 2018; Liu et al., 2021b,c). This is the first study to apply MPS to evaluate the genetic diversity, population structure, and phylogeography of medicinal plant resources. One of the major advances in MPS is the rapidity with which target sequences can be sequenced, as hundreds to thousands of target sequences can be generated over a reasonable timeframe to meet the needs of researchers. In this study, 25,197 target fragments (227 accessions × 111 primer pairs) were generated over 15 days. The cost of MPS is only one-tenth that of Sanger sequencing. In addition, MPS datasets (e.g., dataset-II and III) can have comparable or even higher Pi than datasets of entire plastids (e.g., dataset-I), which means that MPS has high economy. Overall, MPS is an effective approach for studying the genetic diversity, population structure, and phylogeography of medicinal plant resources.

### Phylogeny and Divergence Times of Coptis

*Coptis* is one of the most pharmaceutically important genera worldwide; it is a small genus with only 15 species that are disjunctly distributed from Eastern Asia to North America, and half of the species are distributed in China (Xiang et al., 2018). Clarification of its phylogenetic relationships and divergence time in China is important given that China is one of the centers of its distribution, as well as a region where the medicinal use of *Coptis* is widespread. Previous phylogenetic studies of *Coptis* have been performed using several DNA markers, such as *trnL-F*, *trnD-T*, *trnHpsbA*, *rpoB*, *accD*, and *rbcL* (He et al., 2014; Xiang et al., 2016, 2018). Phylogenetic analysis based on the plastids of *Coptis* revealed similar patterns overall, but the relationships established in our study have stronger support compared with those in previous studies. These data provided us with an opportunity to clarify the relationships within species. *C. quinquesecta* and *C. japonica* were used as outgroups. Species in mainland China formed a monophyletic group and split from *C. japonica* approximately 5.32 Mya (95% HPD: 3.70–6.97). Within this monophyletic clade, *C. teeta* was in the basal position, and a clade formed by *C. deltoidea* and *C. omeiensis* was sister to the clade containing *C. chinensis* var. *chinensis* and *C. chinensis* var. *brevisepala*.

Two variants of *C. chinensis* diverged approximately 2.83 Mya. The subpopulations of *C. chinensis* var. *brevisepala* showed high diversity, and diversification occurred rapidly from 1.30 to 1.57 Mya, which coincides with the period of increased diversification rates of all members of the Ranunculaceae family approximately 2 Mya (Xiang, 2020). Thus, *C. chinensis* var. *chinensis* was expected to show similar levels of diversity among subpopulations, but they were much less diverse. This might stem from founder effects associated with artificial selection.

### Genetic Diversity and Phylogeography of Wild and Cultivated Populations

In breeding, genetic diversity is essential for increasing yields, the adaptation of populations to the environment, and the resistance of populations to pests and diseases. Domestication is an artificial selection process wherein individual plants with desirable properties are bred to develop varieties that can better meet human needs. The action of selection, coupled with the inability to sample all possible variations in the progenitor population, resulted in a decrease in genetic diversity, and this is known as the founder effect. Several studies of crops and horticultural plants have shown that cultivated plants that have diverged from their wild progenitors or relatives in response to human selection show decreased diversity consistent with genetic bottleneck effects (Liu et al., 2020). This apparent loss of genetic diversity likely stems from the occurrence of population bottlenecks during domestication, which has been widely observed in crop species (Mandel et al., 2011; Zhou et al., 2015; Mastretta Yanes et al., 2018).

Medicinal plants might also undergo bottlenecks when subjected to artificial selection. In this study, we showed that a traditional herbal plant that has been cultivated for 700 years (Ccc) has experienced a severe genetic bottleneck and shows extremely low diversity. Compared with Ccb, the Pi of Ccc (0.00032) was only a quarter of that of Ccb (0.00138). The genetic differentiation (Fst) of wild and cultivated populations also indicated that the cultivated population experienced a genetic bottleneck. Previous studies have indicated that *Angelica sinensis*, a medicinal plant that has been cultivated for 2,000 years, has experienced a severe genetic bottleneck (Wang, 2020). By contrast, *Scutellaria baicalensis*, which has only been cultivated for approximately six to seven decades, has not undergone a genetic bottleneck (Yuan et al., 2010). Thus, medicinal plants might undergo bottlenecks following several rounds of artificial selection. Aside from artificial selection and founder effects, admixture has also played an important role in shaping patterns of genetic diversity. Longer periods of cultivation provide greater opportunity for admixture among genetically similar, selected subpopulations, which exacerbates the bottleneck effect.

Phylogeographic analysis demonstrated that the wild and cultivated populations were distinct, which is consistent with the results of the network analysis, PCA, and STRUCTURE analysis. The highly mixed germplasm of Ccc with little population differentiation could be roughly divided into two or three genotypes that were not congruent with their geographic distributions. By comparison, the differences in the genotype composition of the Ccb subpopulations were congruent with geographic differences among Ccb subpopulations, and some gene flow occurred among neighboring populations. This medicinal species provides an ideal model for evaluating the genetic and phylogeographic consequences of domestication on wild and cultivated populations. The lack of genetic diversity in cultivated *C. chinensis* potentially stems from a series of bottlenecks that occurred during its domestication, and this is thought to increase its susceptibility to diseases. Ccc is cultivated over large areas (0.32 million mus) in China, and it is an important resource for many industries and a large segment of the human population. The problems posed by root rot require urgent attention, and the solution to this problem might lie in the effective use of the genetic reservoirs of wild populations.

### Identification and Conservation of Cultivated and Wild *C. chinensis*

Breeding disease-resistant individuals from wild populations is a long process that requires several steps. First, measures to protect wild resources need to be implemented. Ccc and Ccb are difficult to distinguish morphologically in the non-flowering period, especially in the seedling stage; thus, efficient approaches for distinguishing between wild and cultivated *C. chinensis* need to be developed. DNA barcoding could be used to facilitate species identification. Most hot spots were identified through comparisons of various sequences in this study, such as *ycf1*, *trnHpsbA*, *trnK-rps16*, and *rpl32*, which have been widely used as barcodes for discriminating between species or reconstructing phylogenies (Kress and Erickson, 2007; Dong et al., 2014, 2015). These sequences could also be used to develop markers for the identification of wild and cultivated *C. chinensis*. Among the 111 marker pairs generated for MPS, 38 pairs show population-specific variation (SNPs or indels) that could be used for discrimination (Supplementary Table 5).

Genetic diversity is closely associated with the adaptive evolutionary potential and reproductive fitness of populations. The maintenance of genetic diversity is a primary focus of the management of wild populations, especially for endangered species. The genetic diversity of Ccb was high, and the phylogeography of subpopulations was congruent with their geographic distributions. Thus, *in situ* conservation strategies are needed for Ccb. Smaller populations and endangered species often exhibit lower genetic diversity levels (Frankham et al., 2002); however, the diversity of Ccb was high. Habitat destruction and the harvesting of these herbs by humans in recent decades are responsible for its endangered status. When population sizes are low, genetic drift plays a critical role in shaping the genetic structure of populations. The diversity of endangered wild resources may decrease over time, which makes them more susceptible to environmental changes such as changes in climate. Aside from *in situ* conservation and preservation of wild habitat, the population size of wild populations needs to be increased while maintaining their high levels of genetic diversity.

Ccc has experienced a severe genetic bottleneck due to its cultivation. Several measures need to be taken to revert this trend. Seed exchange should be more carefully controlled, and wild genotypes should be utilized in the cultivation process. Thus, we suggest that a government-established germplasm resource nursery with several genotypes be initiated to minimize the inbreeding of Ccc. The decrease in diversity could be mitigated if the government or research institutes played greater roles in regulating the distribution of seedlings rather than the private sector. Overall, the information on the plastids and the hotspot regions obtained in this study are important for protecting wild resources and guiding the establishment of cultivated germplasm resource nurseries.

## Data Availability Statement

The data presented in this study are deposited in the GenBank, accession numbers: OM202488-OM202510.

## Author Contributions

YW and JS did the data analysis and wrote the manuscript. CX and ZZ participated in the experiments. ZZ, PQ, SW, ZX, and MW collected the study materials. QY, LG, and LH conceived and designed the research. All authors read and approved the final manuscript.

## Conflict of Interest

ZX was employed by Lichuan Jianzhuxi Huanglian Cooperative, China. The remaining authors declare that the research was conducted in the absence of any commercial or financial relationships that could be construed as a potential conflict of interest.

## Publisher’s Note

All claims expressed in this article are solely those of the authors and do not necessarily represent those of their affiliated organizations, or those of the publisher, the editors and the reviewers. Any product that may be evaluated in this article, or claim that may be made by its manufacturer, is not guaranteed or endorsed by the publisher.

## Footnotes

1
http://hannonlab.cshl.edu/fastx_toolkit/

2
http://taylor0.biology.ucla.edu/struct_harvest/

## Supplementary Material

Supplementary Figure 1 The boxplot of Pi values and indels in Ccb and Ccc.

Supplementary Figure 2 The maximum likelihood phylogeny of *Coptis*.

Supplementary Figure 3 The delta K value of STRUCTURE analysis.
