# Supplementary figures and images for: Multiplexed Massively Parallel Sequencing of Plastomes Provides Insights Into the Genetic Diversity, Population Structure, and Phylogeography of Wild and Cultivated Coptis chinensis
Source: Front Plant Sci. 2022 Jul 7;13:923600. doi: 10.3389/fpls.2022.923600 (PMC9302112; doi:10.3389/fpls.2022.923600)

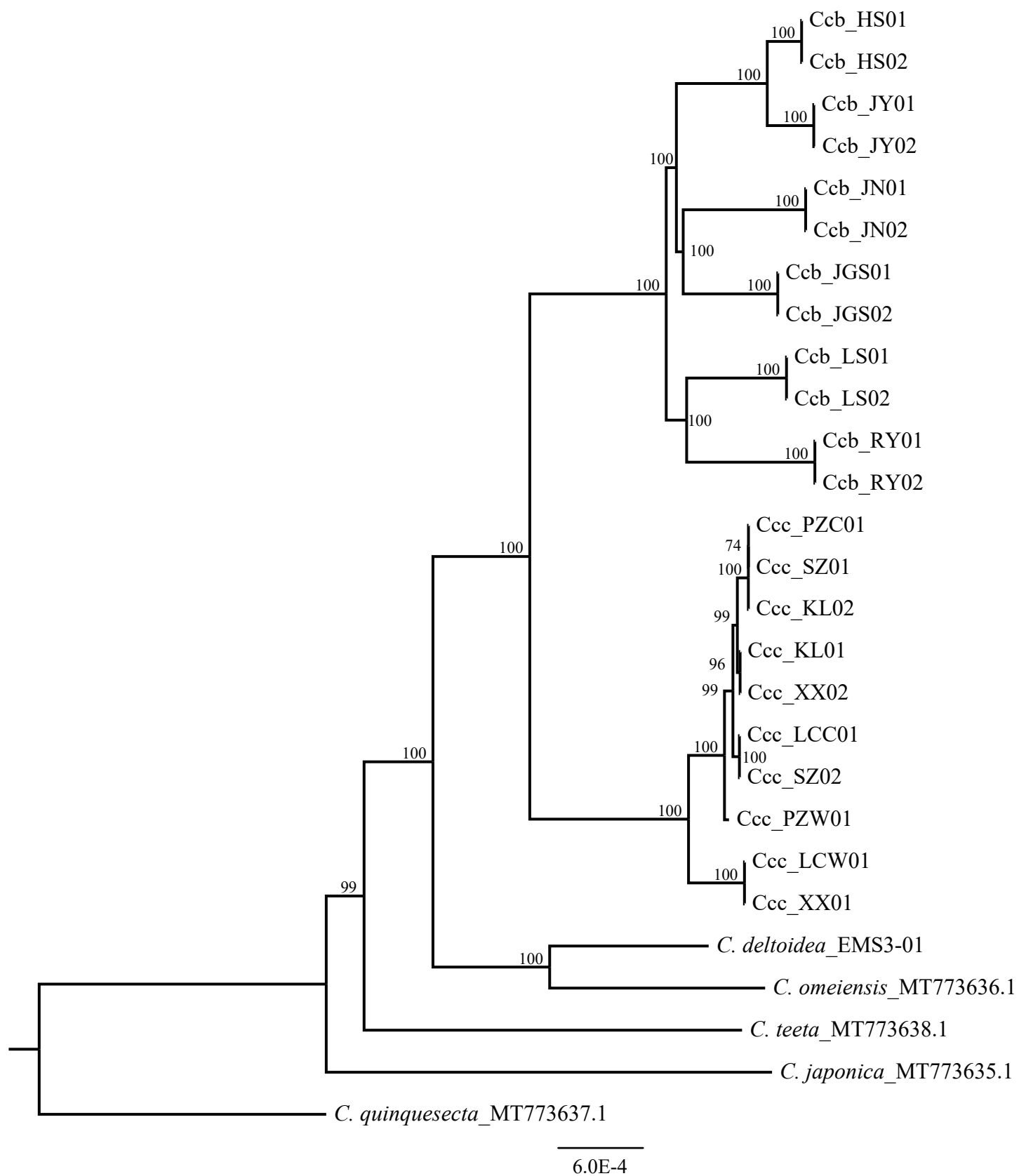

Supplement: Supplementary Figure 1 — The boxplot of Pi values and indels in Ccb and Ccc. [file Data_Sheet_1.PDF]

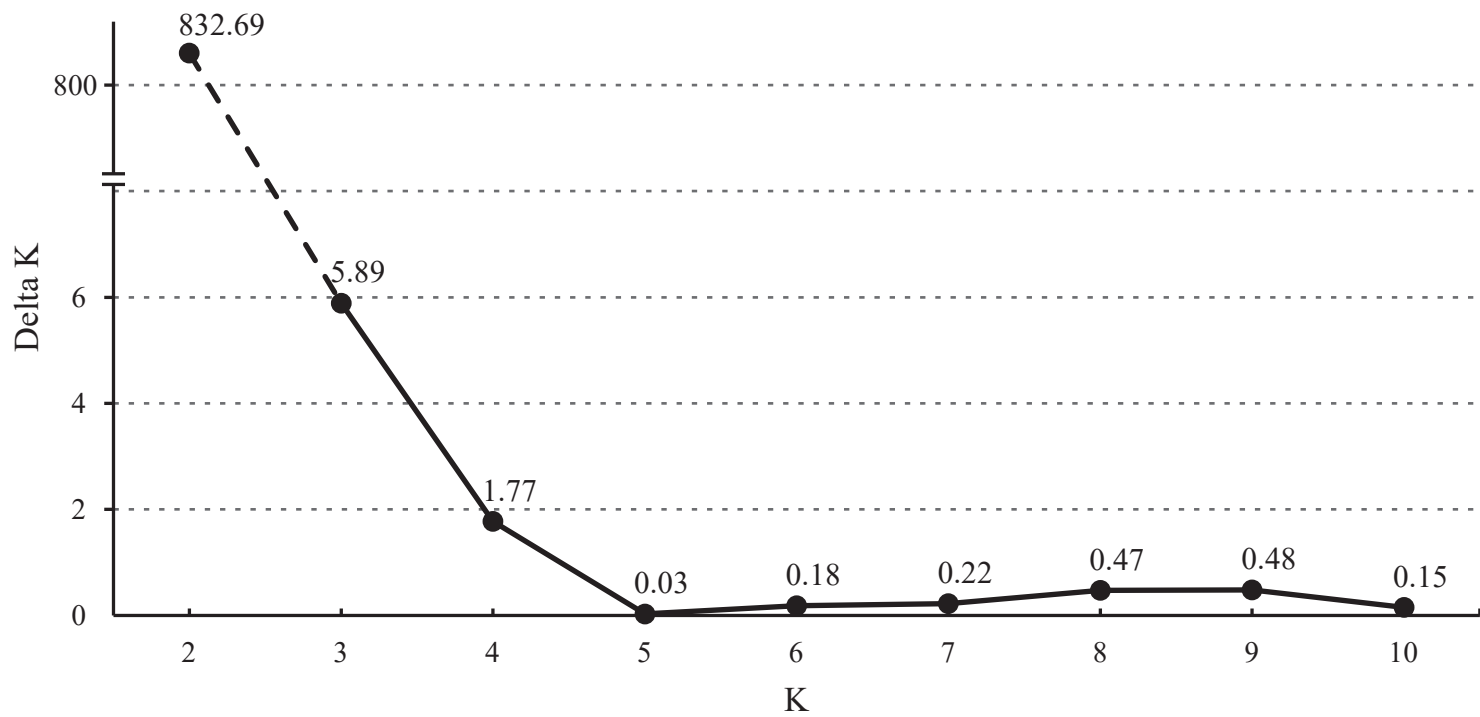

Supplement: Supplementary Figure 2 — The maximum likelihood phylogeny of Coptis. [file Data_Sheet_2.PDF]

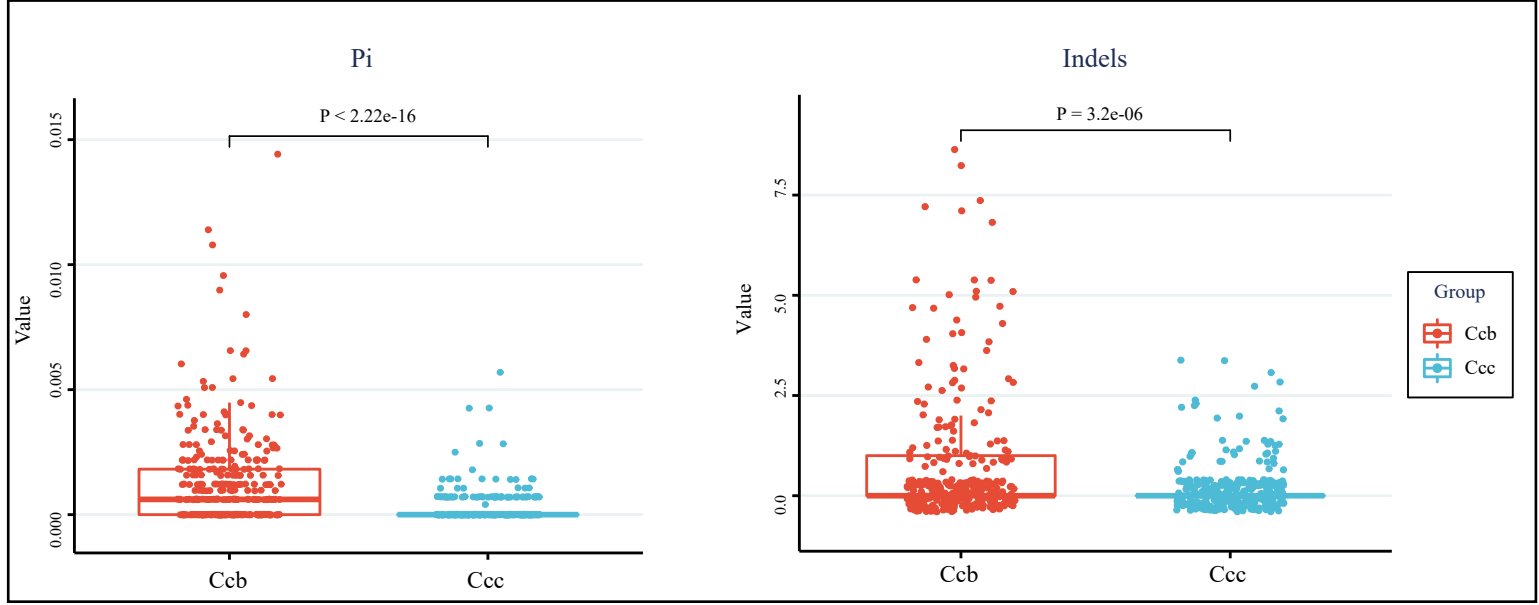

Supplement: Supplementary Figure 3 — The delta K value of STRUCTURE analysis. [file Data_Sheet_3.PDF]
